# A RNA-Sequencing approach for the identification of novel long non-coding RNA biomarkers in colorectal cancer

**DOI:** 10.1038/s41598-017-18407-6

**Published:** 2018-01-12

**Authors:** Atsushi Yamada, Pingjian Yu, Wei Lin, Yoshinaga Okugawa, C. Richard Boland, Ajay Goel

**Affiliations:** 1 Center for Gastrointestinal Research, Translational Genomics and Oncology, Baylor Scott & White Research Institute and Charles A Sammons Cancer Center, Baylor University Medical Center, Dallas, TX 75246 USA; 2 Lab of Genomics and Bioinformatics, Baylor Institute for Immunology Research, Baylor Research Institute, Baylor University Medical Center, 3434 Live Oak Street, Suite 208, Dallas, TX 75204 USA; 3 Department of Therapeutic Oncology, Graduate School of Medicine, Kyoto University, 54 Shogoin-kawara-cho, Sakyo-ku, Kyoto 606-8507 Japan; 4 Department of Gastrointestinal and Pediatric Surgery, Division of Reparative Medicine, Institute of Life Sciences, Graduate School of Medicine, Mie University, 2-174 Edobashi, Tsu, Mie 514-8507 Japan

## Abstract

Long non-coding RNAs (lncRNAs) have been implicated in human pathology, however, their role in colorectal carcinogenesis have not been fully elucidated. In the current study, whole-transcriptome analysis was performed in 3 pairs of colorectal cancer (CRC) and matched normal mucosa (NM) by RNA sequencing (RNA-seq). Followed by confirmation using the Cancer Genome Atlas (TCGA) dataset, we identified 27 up-regulated and 22 down-regulated lncRNAs in CRC. Up-regulation of four lncRNAs, hereby named colorectal cancer associated lncRNA (CRCAL)-1 [AC021218.2], CRCAL-2 [LINC00858], CRCAL-3 [RP11-138J23.1] and CRCAL-4 [RP11-435O5.2], was further validated by real-time RT-PCR in 139 colorectal neoplasms and matched NM tissues. Knockdown of CRCAL-3 and CRCAL-4 in colon cancer cells reduced cell viability and colony formation ability, and induced cell cycle arrest. TCGA dataset supported the associations of CRCAL-3 and CRCAL-4 with cell cycle and revealed a co-expression network comprising dysregulated lncRNAs associated with protein-coding genes. In conclusion, RNA-seq identified numbers of novel lncRNAs dysregulated in CRC. *In vitro* experiments and GO term enrichment analysis indicated the functional relevance of CRCAL-3 and CRCAL-4 in association with cell cycle. Our data highlight the capability of RNA-seq to discover novel lncRNAs involved in human carcinogenesis, which may serve as alternative biomarkers and/or molecular treatment targets.

## Introduction

It is estimated that more than 70% of the human genome is transcribed into RNA, but only up to 2% is translated to proteins; hence, majority of RNA do not serve as a blue print for protein coding genes. RNA molecules which do not encode proteins are called non-coding RNAs (ncRNAs), and historically, most of them in the past were considered as transcriptional noise. Based on their length, ncRNAs are divided into two subgroups; small ncRNAs which are shorter than 200 nucleotides, and long ncRNAs (lncRNAs) that consist of 200 nucleotides or more in length^1–3^. Recent decade has witnessed a growing recognition for the functional relevance of microRNAs, a subgroup of small ncRNAs, as transcriptional repressors by virtue of their interaction with the 3′UTR regions of their downstream target genes. MicroRNAs are known to be involved in cellular differentiation, proliferation and apoptosis, and their dysregulation is known to associate with various human malignancies^4^. In contrast to miRNAs, the biological role of lncRNAs still remain poorly understood, and are an active area of investigation. However, cell-type and developmental time-point specific expression patterns and conserved sequences of lncRNAs raise the possibility that they also possess functional significance in the biological context^1–3^. In fact, functional importance of several lncRNAs have been recently elucidated. For example, HOTAIR recruits polycomb repressive complex 2 to specific target genes, leading to epigenetic re-programming, and its increased expression levels were linked to progression of breast and gastric cancers^5,6^. Another lncRNA, MALAT1, is known to regulate gene expression and alternative splicing, and has been linked to lung and several other human cancers^7^. Nonetheless, majority of lncRNAs have not been well characterized. Given the abundance of lncRNAs existing in human genome, there are perhaps a number of uncharacterized lncRNAs that possibly play key roles in human cancers. Therefore, it would be important to identify and investigate novel lncRNAs involved in human carcinogenesis, which are potentially relevant as biomarkers and/or molecular targets for treatment. RNA sequencing (RNA-seq) is an approach to analyze whole-transcriptome using the next generation sequencing technology, which enables to virtually reconstruct an entire transcriptome, including lncRNAs. With its advantages in terms of a greater dynamic range and the ability to discover novel transcripts, RNA-seq is capable of identifying unknown lncRNAs involved in human pathology^8^. Indeed, RNA-seq technology has been utilized to discover novel lncRNAs in various diseases including prostate^9^, breast^10^, and gastric^11^ cancers.

Colorectal cancer (CRC) is one of the leading causes of cancer-related deaths in the Unites States, and more than 50,000 patients die of this disease annually^12^. Although molecular alterations involved in CRC have been well-known in terms of genetic mutations as well as epigenetic alterations such as DNA methylation^13,14^, the role of lncRNAs and their dysregulation in colorectal carcinogenesis has yet not been fully elucidated.

In the current study, we conducted a systematic and comprehensive identification of novel lncRNAs involved in colorectal carcinogenesis. To this end, we performed RNA-seq using matched cancerous and non-cancerous human colon tissues, followed by the validation of dysregulated lncRNAs by analyzing the Cancer Genome Atlas (TCGA) database (http://cancergenome.nih.gov/) and by real-time RT PCR. The aim of this study was to identify novel lncRNAs associated with colorectal carcinogenesis as alternative biomarkers and/or treatment targets for CRC by using RNA-seq technology.

## Results

### RNA-seq read mapping

A splice-aware mapping solution was implemented for RNA-seq read alignment. The alignment index was built on hg19 genome (including 25 chromosomes and other 68 unplaced contigs) combined with total junction flanking TRANSCRIPTOMIC sequence summarized from GENCODE, EMSEMBLE and REFSEQ annotations. The junction flanking sequence length was defined by the read length subtract 5. Novoalign+ V2.08.01 was used for alignment. Redundant mapping at the same locus for both genome and transcriptome was consolidated as one single hit. The read count for each annotated transcript was then derived from mapped reads by Rsubread^15^. Some of the key statistics of read mapping is shown on Table 1.

**Table 1:** Statistics of reads mapping.

**Numbers of reads**	**CRC samples**	**NM samples**
Raw Illumina HiSeq	37622042	47125659
Unmapped	−3443717	−11039389
Unannotated	−20829243	−22681232
Total mRNA abundance	13349082	13405038

### Identification of dysregulated lncRNAs by RNA-seq

**Figure 1:**
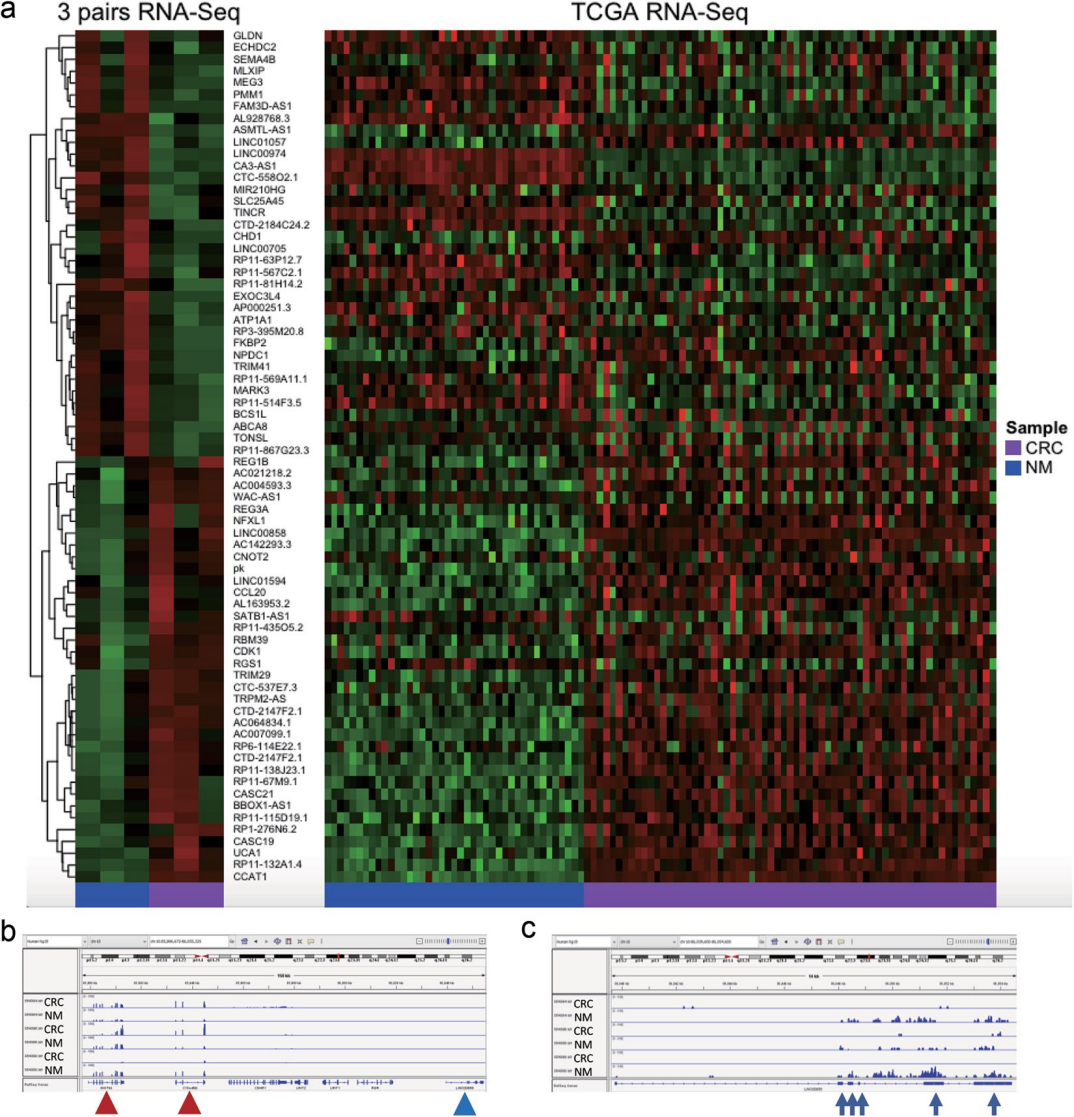
(**a**) Heatmap generated by RNA-seq data from our own sequencing (left) and TCGA (right) datasets showing the distinct expression pattern of lncRNAs in CRC and matched NM tissues. (**b,c**) Sequence information of RNA-seq data was visualized by IGV. Each row indicates paired colonic samples including CRC and matched NM from three patients. The vertical axis represents sequence reads at particular chromosomal position where the scale was large (0–1000) or small (0–5). While the reads for CRCAL-2 [LINC00858] (blue triangle) cannot be recognized with the large scale, nearby protein-coding genes, GHITM and C10orf99 (red triangles), showed abundant read counts (**b**). Although sequence reads for CRCAL-2 (blue arrow heads) was modest, its up-regulation was visible with the small scale (**c**).

A heatmap generated from expression of differentially expressed lncRNAs detected by edgeR on three pairs of matched CRC and NM tissues showed distinct expression patterns of these lncRNAs between CRC and NM tissues. (Fig. 1a) Heatmap plot for the expression of these lncRNAs on TCGA dataset is also shown in Fig. 1a. By analyzing in-house RNA-seq data, 72 lncRNAs were found to be significantly dysregulated in CRC compared to NM tissues. Of these, 27 of 36 up-regulated lncRNAs and 22 of 36 down-regulated lncRNAs were confirmed by TCGA dataset (Table 2). Dysregulation of CCAT1^16^, UCA1^17^, and MEG3^18^ has been previously linked to CRC, while dysregulation of LINC00974^19^ and TRPM2-AS^20^ have been reported in hepatocellular carcinoma and prostate cancer, respectively. In addition, RP11-115D19.1 and TINCR have been functionally associated with certain biological contexts^21,22^. Thus, as a result of in-house RNA-seq and confirmation by TCGA dataset, we could identify 42 novel lncRNAs which have not been previously well documented. When we visualized the RNA-seq reads at each dysregulated lncRNA by using IGV, in contrast to many protein-coding genes showing much larger read counts, majority of dysregulated lncRNAs had very small read counts which were mostly less than 10 (Fig. 1b,c).

**Table 2:** LncRNAs dysregulated in CRC discovered by RNA-seq and confirmed by TCGA.

**Transcript ID**	**Gene ID**	**Gene Symbol**	**logFC**	**p-value**	**FDR**	**lncRNA Type**	**FDR (TCGA)**
ENST00000464746	ENSG00000172016	REG3A	7.812	0.002	0.019	retained_intron	<0.001
ENST00000559321	ENSG00000259485	CTD-2147F2.1	7.719	<0.001	0.007	lincRNA	<0.001
ENST00000521586	ENSG00000253929	CASC21	7.449	0.005	0.015	lincRNA	<0.001
ENST00000436530	ENSG00000225680	AL163953.2	7.157	<0.001	0.019	lincRNA	<0.001
ENST00000413290	ENSG00000224099	AC064834.1	6.954	<0.001	0.015	lincRNA	<0.001
ENST00000514769	ENSG00000251026	RP11-138J23.1	6.55	0.002	0.037	lincRNA	<0.001
ENST00000451622	ENSG00000231172	AC007099.1	6.463	0.002	0.052	antisense	<0.001
ENST00000419196	ENSG00000230234	RP1-276N6.2	6.202	<0.001	0.081	lincRNA	<0.001
ENST00000513572	ENSG00000251095	RP11-115D19.1	6.201	0.001	0.019	antisense	<0.001
ENST00000476618	ENSG00000091436	pk	5.867	0.003	0.093	retained_intron	<0.001
ENST00000456880	ENSG00000230061	TRPM2-AS	5.677	0.003	0.092	antisense	<0.001
ENST00000560314	ENSG00000259485	CTD-2147F2.1	5.671	<0.001	0.093	lincRNA	<0.001
ENST00000521815	ENSG00000254166	CASC19	5.595	<0.001	0.051	lincRNA	<0.001
ENST00000532195	ENSG00000137699	TRIM29	5.541	0.002	0.11	retained_intron	0.018
ENST00000479258	ENSG00000172023	REG1B	5.526	0.002	0.128	retained_intron	<0.001
ENST00000510419	ENSG00000249942	AC142293.3	5.309	0.002	0.146	antisense	<0.001
ENST00000500112	ENSG00000247844	CCAT1	4.9	0.004	0.019	lincRNA	<0.001
ENST00000446246	ENSG00000235669	AC004593.3	4.326	0.001	0.151	antisense	<0.001
ENST00000531363	ENSG00000254560	BBOX 1-AS1	4.19	0.003	0.16	antisense	<0.001
ENST00000445083	ENSG00000225328	LINC01594	4.071	0.003	0.188	antisense	<0.001
ENST00000456253	ENSG00000228956	SATB1-AS1	4.044	0.001	0.166	antisense	0.007
ENST00000419422	ENSG00000232445	RP11-132A1.4	4.037	<0.001	0.091	antisense	<0.001
ENST00000415469	ENSG00000229404	LINC00858	3.708	<0.001	0.166	lincRNA	<0.001
ENST00000498352	ENSG00000090104	RGS1	3.646	0.003	0.155	retained_intron	<0.001
ENST00000397381	ENSG00000214049	UCA1	3.602	<0.001	0.146	lincRNA	<0.001
ENST00000433644	ENSG00000237857	RP11-435O5.2	3.354	<0.001	0.188	lincRNA	0.042
ENST00000377722	ENSG00000204876	AC021218.2	3.134	<0.001	0.192	lincRNA	<0.001
ENST00000497872	ENSG00000253701	AL928768.3	−3.205	0.002	0.146	lincRNA	<0.001
ENST00000522615	ENSG00000254042	CTC-558O2.1	−3.341	0.002	0.16	antisense	<0.001
ENST00000531791	ENSG00000162241	SLC25A45	−3.344	0.003	0.188	retained_intron	0.045
ENST00000488268	ENSG00000121310	ECHDC2	−3.354	0.004	0.18	retained_intron	0.002
ENST00000548722	ENSG00000257194	RP11-567C2.1	−3.448	0.004	0.188	lincRNA	<0.001
ENST00000452922	ENSG00000224081	LINC01057	−3.459	<0.001	0.166	lincRNA	<0.001
ENST00000524052	ENSG00000253549	CA3-AS1	−3.712	0.004	0.145	antisense	<0.001
ENST00000456403	ENSG00000226629	LINC00974	−3.771	0.002	0.143	lincRNA	<0.001
ENST00000428573	ENSG00000226862	RP11-569A11.1	−3.89	<0.001	0.151	antisense	0.005
ENST00000464125	ENSG00000244383	FAM3D-AS1	−4.097	<0.001	0.142	antisense	<0.001
ENST00000536094	ENSG00000251301	RP11-81H14.2	−4.101	<0.001	0.197	lincRNA	<0.001
ENST00000448587	ENSG00000223573	TINCR	−4.111	0.002	0.057	lincRNA	<0.001
ENST00000488733	ENSG00000163399	ATP1A1	−4.144	0.004	0.145	retained_intron	0.012
ENST00000463617	ENSG00000100417	PMM1	−4.152	0.003	0.185	retained_intron	0.004
ENST00000464150	ENSG00000186417	GLDN	−4.161	0.003	0.152	retained_intron	<0.001
ENST00000533203	ENSG00000255186	RP11-514F3.5	−4.199	<0.001	0.162	sense_intronic	<0.001
ENST00000559274	ENSG00000075413	MARK3	−4.243	<0.001	0.161	retained_intron	0.008
ENST00000529171	ENSG00000254718	CTD-2184C24.2	−4.351	0.003	0.168	antisense	<0.001
ENST00000433071	ENSG00000237594	AP000251.3	−4.826	<0.001	0.093	antisense	<0.001
ENST00000501708	ENSG00000245156	RP11-867G23.3	−4.883	0.004	0.087	lincRNA	0.021
ENST00000427901	ENSG00000235523	RP11-63P12.7	−5.119	<0.001	0.061	lincRNA	<0.001
ENST00000398460	ENSG00000214548	MEG3	−5.238	<0.001	0.151	lincRNA	<0.001

### Validation of dysregulated lncRNAs in colorectal tumors by real-time RT-PCR

**Figure 2:**
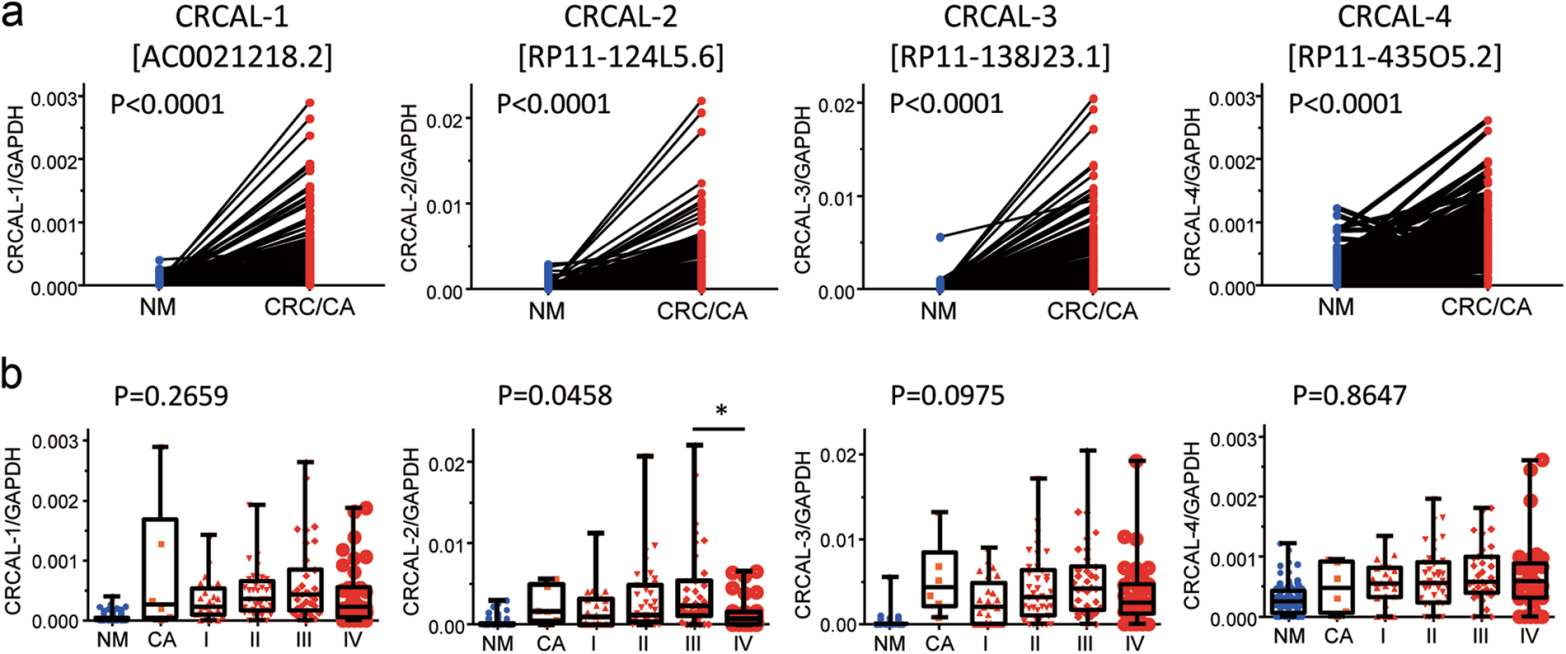
(**a**) Significant increase in transcripts levels of four lncRNAs, CRCAL-1 [AC021218.2], CRCAL-2 [LINC00858], CRCAL-3 [RP11-138J23.1], and CRCAL-4 [RP11-435O5.2], were validated by real-time RT-PCR in 139 colorectal tumors including 134 CRCs and 5 CAs. (**b**) Levels of CRCAL-1, CRCAL-3 and CRCAL-4 were not significantly different, while CRCAL-2 levels differed with marginal significance among CAs and stage I to IV CRCs. By the Steel-Dwass test, levels of CRCAL-2 were significantly higher in stage III than in stage IV CRCs (*P < 0.05).

To validate the dysregulated expression of lncRNAs found by RNA-seq in more colorectal tumors, we performed real-time RT-PCR to examine the levels of four lncRNAs, AC021218.2, LINC00858, RP11-138J23.1 and RP11-435O5.2, in 139 colorectal tumors including 134 CRCs and 5 colorectal adenomas (CAs), and 139 matched normal mucosae (NM) tissues. In line with the results of RNA-seq, levels of four lncRNAs examined were significantly higher in colorectal tumors compared to matched NM tissues (Fig. 2a). Given the association of these novel lncRNAs with CRC, we hereby name them as ColoRectal Cancer Associated LncRNAs (CRCAL)-1 [AC021218.2], CRCAL-2 [LINC00858], CRCAL-3 [RP11-138J23.1], and CRCAL-4 [RP11-435O5.2]. Levels of CRCALs did not change among adenoma and stage I-IV CRCs, except for CRCAL-2 which showed significantly lower levels in stage IV compared to stage III CRCs. Thus, no stepwise increase during the tumor progression was observed (Fig. 2b).

### Knockdown of CRCAL-3 and CRCAL-4 in colon cancer cells

**Figure 3:**
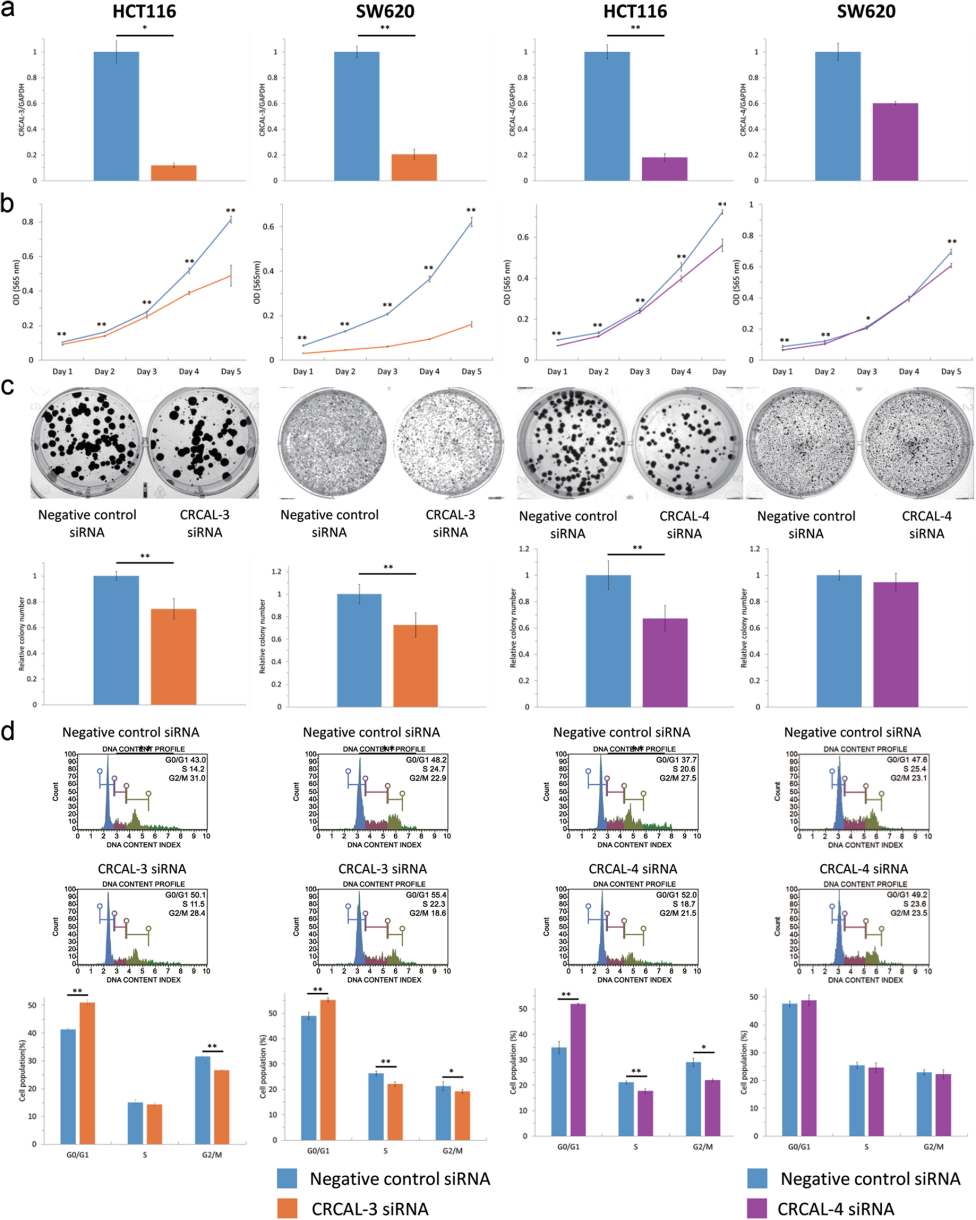
Knockdown of CRCAL-3 and CRCAL-4 by siRNA in HCT116 and SW620 cells. (**a**) Transfection of siRNA for CRCAL-3 or CRCAL-4 induced significant repression of respective lncRNA transcripts. (**b**) MTT assay showed significant inhibition of cell viability after either CRCAL-3 or CRCAL-4 knockdown. (**c)** CRCAL-3 knockdown inhibited colony formation ability in HCT116 and SW620 cells. Colony formation was inhibited by CRCAL-4 knockdown in HCT116 but not in SW620 cells. (**d)** Cell cycle analyses showed the G0/G1 arrest in HCT116 and SW620 cells after CRCAL-3 knockdown and in HCT116 cells after CRCAL-4 knockdown. (*P < 0.05, **P < 0.01).

To gain further insight into whether these dysregulated lncRNAs have any functional role in CRC, we performed knockdown of CRCAL-3 and CRCAL-4 by transfecting siRNAs in colon cancer cells, HCT116 and SW620. As expected, levels of both CRCAL-3 or CRCAL-4 decreased significantly after transfection of siRNA specific for respective lncRNA, confirming their successful knockdown (Fig. 3a). CRCAL-3 knockdown resulted in decreased cell viability by MTT assay, reduced colony formation ability, and cell cycle arrest at G0/G1 in both cell lines. Knockdown of CRCAL-4 showed similar effects in HCT116 cells, however, it caused minimal inhibition of cell viability but no obvious effects on colony formation ability nor cell cycle progression in SW620 cells (Fig. 3b–d).

### Analyses of CRCAL-3 and CRCAL-4 expression in TCGA dataset

**Figure 4:**
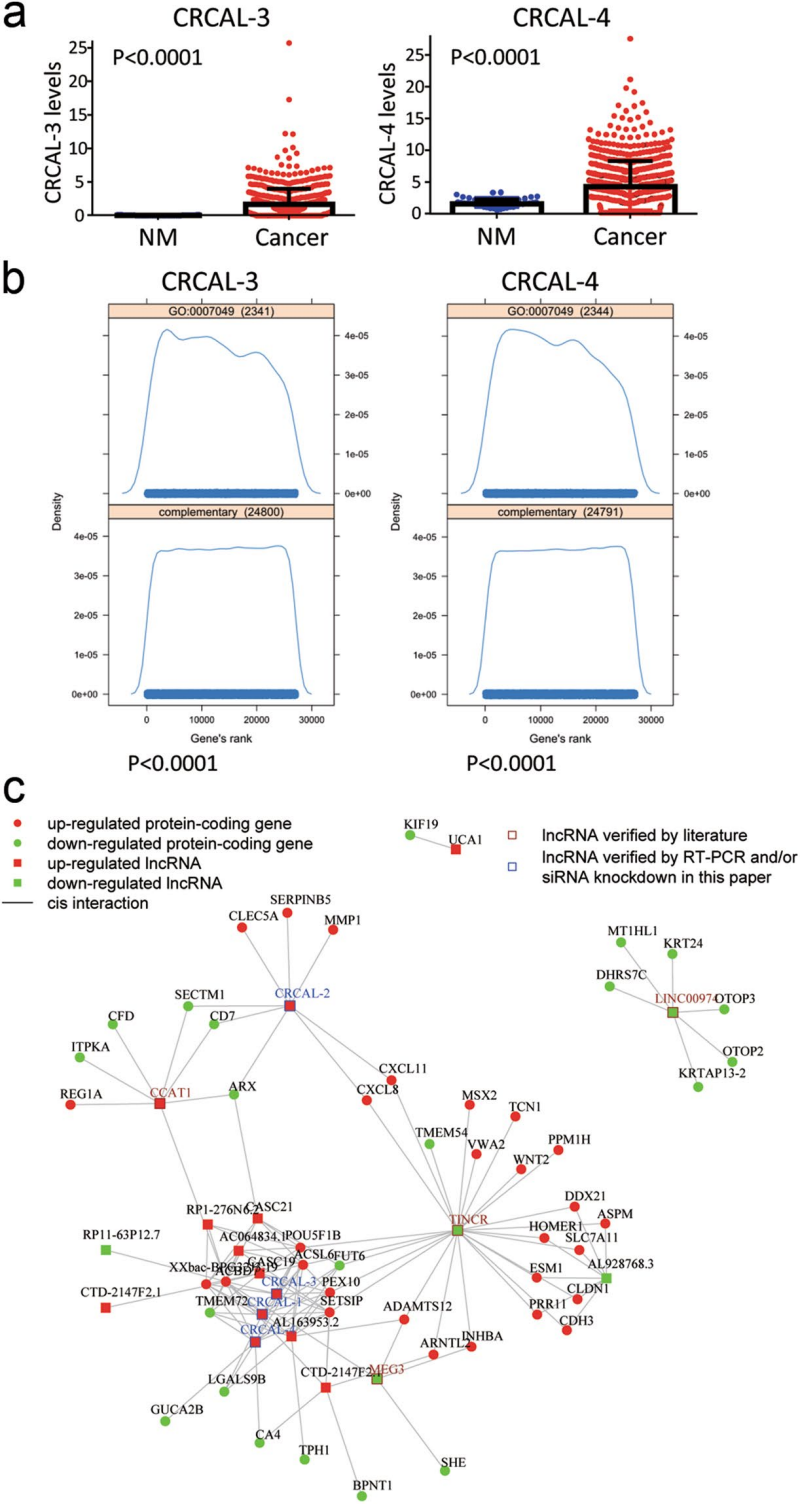
Analyses of RNA-seq from TCGA dataset in 682 colon cancers and 41 NMs. (**a**) Expression levels of both CRCAL-3 and CRCAL-4 were significantly higher in colon cancer than in NM tissues. (**b**) GO term enrichment analysis confirmed the significant association of either CRCAL-3 or CRCAL-4 with cell cycle. (**c**) Differentially expressed lncRNAs and protein-coding genes from RNA-seq data formed a co-expression network in colon cancer tissues. Known and novel lncRNAs associated with colon cancer were identified.

To further validate the significance of CRCAL-3 and CRCAL-4 in colorectal carcinogenesis using an independent dataset, we again utilized RNA-seq data of 682 colon cancers and 41 normal tissues from TCGA database. First, we compared the expression levels of CRCAL-3 and CRCAL-4 between colon cancers and normal tissues, and as mentioned above, we confirmed the significant up-regulation of two lncRNAs in colon cancers (Fig. 4a). Since we observed the relation of two lncRNAs to cell cycle by *in vitro* knockdown experiments, we next analyzed the association of either CRCAL-3 or CRCAL-4 with cell cycle. We performed the GO term enrichment analysis (Fig. 4b), and found that the ranks of correlation between CRCAL-3 (CRCAL-4) and cell cycle (GO:0007049) genes were significantly higher than those between CRCAL-3 (CRCAL-4) and background genes, which indicates the significant association of these lncRNAs with cell cycle. Finally, we drew a co-expression network comprised of vertices, which represent differentially expressed lncRNAs or protein-coding genes from RNA-seq datasets, and edges, which represent the co-expression (measured by Pearson’s correlation) of lncRNAs and protein-coding genes in colon cancer tissues. As shown in Fig. 4c, some of hub lncRNAs in the network have been verified either by literatures (CCAT1 in^16^, MEG3 in^18^, LINC00974^19^ and TINCR in^21,22^) or by RT-PCR as well as *in vitro* knockdown in our experiments (CRCAL-1, CRCAL-2, CRCAL-3 and CRCAL-4). There are large overlaps between the target protein-coding genes of CRCAL-1, CRCAL-3 and CRCAL-4, therefore these three lncRNAs may function together in a pathway that is different than that of CRCAL-2.

## Discussion

In the current study, we performed a systematic and comprehensive identification of dysregulated lncRNAs in CRC, and found four lncRNAs, CRCAL-1, CRCAL-2, CRCAL-3 and CRCAL-4, as novel lncRNAs involved in colorectal carcinogenesis. First, by using RNA-seq technology followed by analysis of TCGA dataset, we discovered distinct lncRNA expression patterns between CRC and adjacent NM tissues. Furthermore, we could identify a number of candidate lncRNAs that were dysregulated in CRCs. Looking into RNA-seq data, the read depths for these lncRNAs were generally small. In fact, maximum read counts were less than 10 in most of our candidate lncRNAs. The small read depth can be caused by technical issues regarding RNA-seq. However, considering that the read depths of many protein-coding genes were much larger, the low read depths of lncRNAs is more likely to reflect the limited abundance of lncRNA molecules compared to those of mRNAs of protein-coding genes. In fact, out of 72 lncRNAs found by our in-house RNA-seq analysis, we were able to confirm the dysregulation of 49 lncRNAs by utilizing TCGA dataset in 682 colon cancers. Moreover, we could further validate the up-regulation of four CRCALs in an independent cohort of 139 pairs of colorectal tumors and adjacent mucosa by means of real-time RT-PCR. Thus, although the low read depths made it difficult to distinguish lncRNA sequence from artifacts, and we used as little as three pairs of CRC and NM tissues for initial discovery by RNA-seq, our bioinformatics analyses enabled successful identification of novel lncRNAs associated with colorectal carcinogenesis. Collectively, RNA-seq approach appears to be a useful technology to discover novel lncRNAs that are involved in CRCs, and perhaps in other human cancers.

Although we identified novel CRCALs up-regulated in CRCs, their functional roles have not been previously elucidated. Since expression levels of all four CRCALs were elevated in CA tissues, and no obvious stepwise increase during the course of CRC progression was observed, these might be involved in the very early steps of neoplastic process. Given this early dysregulation of CRCALs in colorectal carcinogenesis, these noncoding RNAs might serve as potential biomarkers for early detection of CRC. Therefore, in the future studies, it is important to determine whether their dysregulation is also detectable by using noninvasively collected samples such as blood. In addition, it should be further investigated if CRCRLs have any association with known biomarkers of CRC such as microsatellite instability (MSI). In colon cancer cells, we observed that knockdown of CRCAL-3 and CRCAL-4 reduced cell viability and colony formation ability, and induced cell cycle arrest at G0/G1 phase. The associations of these lncRNAs with cell cycle were further supported by GO term enrichment analysis performed by using TCGA dataset, indicating the functional relevance of CRCAL-3 and CRCAL-4 in CRC. By analyzing the TCGA data, we also found a strong association between the expression levels of dysregulated lncRNAs and those of protein-coding genes forming a co-expression network. Although we only focused on functional relevance of two CRCALs, such co-expression network associating multiple lncRNAs and protein-coding genes may play important roles in driving colorectal neoplasia.

In conclusion, we conducted a systematic and comprehensive study to identify novel lncRNAs involved in colorectal carcinogenesis by using RNA-seq technology. We identified CRCAL-1, CRCAL-2, CRCAL-3 and CRCAL-4 as up-regulated lncRNAs in CRC in two independent cohorts. Functional relevance of CRCAL-3 and CRCAL-4 related to cell cycle was suggested by *in vitro* experiments as well as by GO term enrichment analysis in the TCGA dataset. Our data highlight the capability of RNA-seq technology to discover novel lncRNAs involved in human carcinogenesis, which may serve as alternative biomarkers and/or molecular treatment targets for human cancers.

## Methods

### Patients and clinical specimens

A total of 278 colorectal tissue specimens were analyzed in this study. These human tissues consisted of 134 CRCs, 5 CA and 139 matched adjacent NM which were obtained at the Mie University Hospital between January 2005 and July 2011. Characteristics of study subjects are summarized in Supplementary Table S1. Tissue samples were collected during surgery and immediately stabilized by immersing them in RNAlater solution (Life Technologies, Carlsbad, CA, U.S.A), which were then stored at −80 °C until RNA extraction. Written informed consent was obtained from all study subjects, and the study protocol was approved by the Institutional Review Board of the Baylor Scott & White Research Institute, and all experiments were performed in accordance with relevant guidelines and regulations.

### Cell culture and RNA interference mediated knockdown of lncRNAs

Colon cancer cell lines, HCT116 and SW620, were purchased from ATCC and were grown in Iscove’s modified Dulbecco’s medium (Invitrogen, Carlsbad, CA, U.S.A., catalog number 12440061) with 10% fetal bovine serum and 1% penicillin and streptomycin (Sigma-Aldrich, St. Louis, MO, U.S.A.), and maintained in a humidified 5% CO_2_ incubator at 37 °C. Custom designed siRNAs (Silencer Select siRNA) for CRCAL-3 [RP11-138J23.1] and CRCAL-4 [RP11-435O5.2] and negative control siRNAs (Silencer Select Negative Control #1 siRNA) were purchased from Ambion (Foster City, CA, U.S.A.). Sequence of siRNAs specific for CRCAL-3 and CRCAL-4 are summarized in Supplementary Table S2. Cells were seeded at a density of 2 × 10^5^ cells per well in 6-well plates and cultured for 24 hours. Thereafter, each siRNA with the final concentration of 30 μM was transfected using the Lipofectamine® RNAiMAX Transfection Reagent (Invitrogen, catalog number 13778075). The cells were incubated for 48 hours and then subjected to RNA extraction or to additional experiments.

### RNA extraction

RNA was extracted using miRNeasy Mini Kit (QIAGEN, Hilden, Germany) according to the manufacturer’s instruction. Briefly, 700 μL of QIAzol was added to samples and homogenized with a TissueLyser LT (QIAGEN) for RNAlater immersed tissues or by vortexing for 1 minute for cultured cells. After incubation of the homogenate for 5 minutes at room temperature, 140 μL chloroform was added and centrifuged at 12,000 g and at 4 °C for 15 minutes. Thereafter, transfer the upper aqueous phase to a new tube, and total RNA was extracted and eluted in 60 μL of RNase-free water using QIAcube (QIAGEN).

### RNA sequencing (RNA-seq)

RNA from 6 tissue samples including 3 CRCs and 3 matched adjacent NM were utilized for RNA-seq. RNA-seq was performed by Illumina HiSeq. 2000 platform. RNA-seq dataset was visualized by using the Integrative Genomics Viewer (IGV)^23^.

### Real-time RT-PCR

Expression levels of 4 lncRNAs were examined by real-time RT-PCR in 139 pairs of colorectal tumors (134 CRCs and 5 CAs), and matched NM tissues. Primers used in this study are summarized in Supplementary Table S3. Reverse transcription was performed using 0.5 μg of total RNA with random hexamers and by Advantage RT-for-PCR Kit (Clontech, Mountain View, CA, U.S.A., catalog number 639506). Real-time PCR was conducted using Fast SYBR Green Master Mix (Applied Biosystems, Foster City, CA, U.S.A), and performed in duplicate on the StepOne Plus system (Applied Biosystems). Cycle threshold (Ct) values were calculated using StepOne Software v2.3 (Applied Biosystems), and the expression levels of lncRNAs were normalized to those of GAPDH and determined by the 2-∆Ct method in which ∆Ct were calculated as follows: ∆Ct = Ct (lncRNA of interest) − Ct (GAPDH).

### Cell viability, cell cycle, and colony formation assays

Cell viability was determined using the MTT (3-(4,5-dimethylthiazole-2-yl)-2,5-diphenyl tetrazolium bromide) assay (Sigma-Aldrich, catalog number M5655) as previously described^24^. Cells were transfected with either siRNA specific for CRCAL-3, CRCAL-4 or negative control siRNA, and re-plated at 5 × 10^3^ in 96-well plates after 48 hours incubation. Optical density (OD) was determined at 565 nm by spectrophotometry (Infinite M200 PRO, Tecan, Männedorf, Switzerland) at 24, 48, 72, 96 and 120 hours after re-plating. Cell cycle analysis was conducted 96 hours after siRNA transfection using the Cell Cycle Assay Kit (Merck Millipore, Billerica, MA, U.S.A., catalog number MCH100106) and the Muse Cell Analyzer (Merck Millipore) according to the manufacturer’s instructions. For colony formation assays, cells were re-plated at 5 × 10^2^ in 6-well plates 72 hours after siRNA transfection. About 14 days later, cells were fixed and then stained by 0.5% crystal violet (Sigma-Aldrich, catalog number HT90132), and the number of colonies was counted using the GeneTools image analysis software (Syngene, Frederick, MD, U.S.A.). All experiments were conducted in at least two independent times.

### TCGA data analyses

RNA-seq data for colon cancer (682 samples) as well as normal colon tissues (41 samples) were downloaded from Cancer Genomics Hub. Differential gene expression analysis was performed on this dataset to verify the differentially expressed genes found from 3-pair RNA-Seq dataset. Large sample size of TCGA dataset enables us to perform correlation-based gene set enrichment analysis. Pearson’s correlation test between lncRNA of interest and other gene was performed and ranked. GO term enrichment analysis was performed on the ranked gene set through topGO (http://www.bioconductor.org/packages/release/bioc/html/topGO.html). TCGA dataset also enables us to build transcriptional co-expression network. The edges of the co-expression network were chosen based on the correlation between lncRNA and protein-coding gene across TCGA colon cancer samples (0.5% of strongest negative correlation and 0.5% of strongest position correlation). The vertices of the co-expression network were chosen based on differentially expressed lncRNAs and protein-coding genes on both TCGA RNA-Seq dataset (FDR < 0.05) and 3-pair RNA-Seq dataset (FDR < 0.2).

### Statistical analysis

Differential gene expression of RNA-seq data was analyzed by edgeR^25^. Read counts were fitted into Negative Binomial distribution with two GLM models: one model has only one regressor (patient), whereas the other model has two regressors (patient and treatment; CRC or NM). And then a pairwise comparison between matched CRC and NM was performed using likelihood ratio test between the two GLM models. Genes with a false discovery rate (FDR) less than 0.20 on 3 pairs RNA-Seq dataset (FDR < 0.05 on TCGA dataset) were considered to be significantly dysregulated. Statistical analyses to compare the lncRNA levels measured by real-time RT-PCR were carried out using JMP^®^ 10 (SAS institute Inc., Cary, NC, U.S.A.). The Wilcoxon signed-rank test was conducted for the comparison between matched colorectal tumor and NM tissues. The Kruskal-Wallis test was performed to compare lncRNA levels among tumor stages, and the Steel-Dwass test was used to perform all-paired multiple comparisons. All experimental data were presented as mean ± SD, and the Student’s t-test was used to compare the differences between groups. All P-values were two-sided and a P-value of <0.05 was considered significant.

### Data availability

The datasets generated during and/or analysed during the current study are available in the GEO database at GSE104178.

## Electronic supplementary material

Supplementary data
