# Supplementary material for: A RNA-Sequencing approach for the identification of novel long non-coding RNA biomarkers in colorectal cancer
Source: Sci Rep. 2018 Jan 12;8:575. doi: 10.1038/s41598-017-18407-6 (PMC5766599; doi:10.1038/s41598-017-18407-6)
Supplement: Supplementary file 1 — Supplementary data [file 41598_2017_18407_MOESM1_ESM.pdf]

Supplementary Information

A RNA-Sequencing approach for the identification of novel long non-coding RNA biomarkers in colorectal cancer

Atsushi Yamada, Pingjian Yu, Wei Lin, Yoshinaga Okugawa, C. Richard Boland and Ajay Goel

Supplementary Table S1. Characteristics of study subjects.

**Age**	
Median (range)	69 (34-92)
**Gender**	
Male (%)	83 (59.3)
Female (%)	57 (40.7)
**Location**	
Right (%)	45 (32.4)
Left (%)	94 (67.6)
**Tumor size (mm)**	
Median (range)	40 (10-125)
**Stage (%)**	
Adenoma	5 (3.6)
I	25 (18.0)
II	39 (28.1)
III	35 (25.2)
IV	35 (25.2)

Supplementary Table S2. siRNA sequence used in this study.

**CRCAL-3**	Sense	GGC AGA AAC GUG UUG UAU Utt
[RP11-138J23.1]	Antisense	AAU ACA ACA CGU UUC UGC Ctt
**CRCAL-4**	Sense	GUG UGU UCA UAU GAC GAA Att
[RP11-435O5.2]	Antisense	UUU CGU CAU AUG AAC AGA Cag

Supplementary Table S3. PCR Primers used in this study.

**CRCAL-1**	Forward	GGG AGA AAG GGG AGG TTC AA
[AC0021218.2]	Reverse	AAG AGA CAC CCA CAG ACC TG
**CRCAL-2**	Forward	GAA CAG CTC CGG TCT ACA GC
[RP11-124L5.7]	Reverse	GTC TCC ATG CCT TGC AAT TT
**CRCAL-3**	Forward	GAC CCG ATT TTC CAG TTT CAA AC
[RP11-138J23.1]	Reverse	TGC CTT CTA AAC AGC ATG CAA AT
**CRCAL-4**	Forward	GAT GCC AGC AGA CAG CTT TT
[RP11-435O5.2]	Reverse	AAC TGG GAG GAA CCA AGT CA
